# Experimental Approach for the Uncertainty Assessment of 3D Complex Geometry Dimensional Measurements Using Computed Tomography at the mm and Sub-mm Scales

**DOI:** 10.3390/s17051137

**Published:** 2017-05-16

**Authors:** Roberto Jiménez, Marta Torralba, José A. Yagüe-Fabra, Sinué Ontiveros, Guido Tosello

**Affiliations:** 1 Centro Universitario de la Defensa, A.G.M. Carretera Huesca s/n, 50090 Zaragoza, Spain; rjimenez@unizar.es; 2 I3A, Universidad de Zaragoza, María de Luna 3, 50018 Zaragoza, Spain; jyague@unizar.es; 3 Department of Industrial Engineering, Autonomous University of Baja California, 14418 Tijuana, Mexico; sinue.ontiveros@uabc.edu.mx; 4 Department of Mechanical Engineering, Technical University of Denmark, DK-2800 Kgs. Lyngby, Denmark; guto@mek.dtu.dk

**Keywords:** micro-computed tomography, complex geometry, dimensional measurement, uncertainty, maximum permissible error

## Abstract

The dimensional verification of miniaturized components with 3D complex geometries is particularly challenging. Computed Tomography (CT) can represent a suitable alternative solution to micro metrology tools based on optical and tactile techniques. However, the establishment of CT systems’ traceability when measuring 3D complex geometries is still an open issue. In this work, an alternative method for the measurement uncertainty assessment of 3D complex geometries by using CT is presented. The method is based on the micro-CT system Maximum Permissible Error (MPE) estimation, determined experimentally by using several calibrated reference artefacts. The main advantage of the presented method is that a previous calibration of the component by a more accurate Coordinate Measuring System (CMS) is not needed. In fact, such CMS would still hold all the typical limitations of optical and tactile techniques, particularly when measuring miniaturized components with complex 3D geometries and their inability to measure inner parts. To validate the presented method, the most accepted standard currently available for CT sensors, the Verein Deutscher Ingenieure/Verband Deutscher Elektrotechniker (VDI/VDE) guideline 2630-2.1 is applied. Considering the high number of influence factors in CT and their impact on the measuring result, two different techniques for surface extraction are also considered to obtain a realistic determination of the influence of data processing on uncertainty. The uncertainty assessment of a workpiece used for micro mechanical material testing is firstly used to confirm the method, due to its feasible calibration by an optical CMS. Secondly, the measurement of a miniaturized dental file with 3D complex geometry is carried out. The estimated uncertainties are eventually compared with the component’s calibration and the micro manufacturing tolerances to demonstrate the suitability of the presented CT calibration procedure. The 2U/T ratios resulting from the validation workpiece are, respectively, 0.27 (VDI) and 0.35 (MPE), by assuring tolerances in the range of ± 20–30 µm. For the dental file, the E_N_ < 1 value analysis is favorable in the majority of the cases (70.4%) and 2U/T is equal to 0.31 for sub-mm measurands (L < 1 mm and tolerance intervals of ± 40–80 µm).

## 1. Introduction

The manufacturing of micro three-dimensional components with complex geometries increasingly requires high accuracy metrological tools for process optimization and product tolerance verification in the 10^0^–10^1^ μm range. Sub-μm measurement resolution and repeatability, with combined expanded uncertainty in the single-digit micrometer range down to 1 μm, are to be obtained for effective verification of 3D complex micro geometries.

For this purpose, several contact and non-contact micro metrological techniques are currently available. Tactile micro-coordinate measuring machines (μCMM) can provide the required metrological performances [1,2], but are limited in terms of measuring capability because of: mechanical filtering of the probe, accessibility and minimum measurable feature size due to the probe and stylus dimensions, measuring point density, measuring time, and deformation of high aspect ratio structures under measurement and of soft substrate materials due to the probing force. Non-contact measuring instruments based on optical techniques [3] (e.g., optical CMM [4], confocal and focus variation microscopes [5], coherence scanning interferometers [6], fringe detection [7], and photogrammetry [8]), are capable of meeting the metrological requirements for metrology of 3D micro components, but have limitations both in measuring vertical walls and high aspect ratio structures, and accessing out-of-sight features. When a 3D geometry is measured by these systems, their measurement uncertainty results are influenced by, e.g., changes in the effective focus of the lens and needed motions of the workpiece. Therefore, in many cases a reliable characterization of 3D geometries is particularly difficult with these systems, especially for those with freeform surfaces. In addition, both contact and non-contact techniques share a further limitation: they are not able to measure inaccessible internal features.

A viable solution to these limitations is the use of micro-Computed Tomography (μCT or micro-CT) for geometrical coordinate measurements [9,10]. Micro-CT is a non-contact imaging technique that can provide a densely populated 3D scanning point cloud of an object, allowing the measurements of both external and otherwise non-accessible internal structures, features, and multi-material components [11,12]. For this reason, CT poses an alternative to other measuring techniques. However, the challenging issues for the complete acceptance of computer tomography for metrology purposes are the numerous and complex factors which influence the μCT performance, and the lack of accepted test procedures and standards. Both limitations are directly related to the measurement uncertainty evaluation, needed to perform a reliable establishment of traceability.

Particularly for miniaturized three dimensional complex geometries (as in the case considered in this research, i.e., a dental file that presents a complex helix geometry with variable diameter, helix angle, and pitch along the component, as well as a variable sub-mm diameter), µCT represents a suitable solution for these measuring tasks. However, due to the intrinsic complexity of computed tomography, problems for a reliable uncertainty assessment of CT measurements arise. In recent years, different approaches have been presented. This work will firstly review the current state-of-the-art of methods and international standards for the establishment of the traceability of measuring systems equipped with CT sensors. The most accepted procedure based on the assessment of the measurement uncertainty by means of the calibrated workpiece using CT [13] is not suitable in the case of components with 3D complex geometries, as arguably there is a lack of a traceable measuring technology that can be employed for such measuring tasks and that provides reference calibration data. Furthermore, the VDI/VDE 2630 Part 2.1 guideline [13] is only developed for external geometries, so that the main metrological advantage of X-ray computed tomography, i.e., its ability to measure inner geometries, is not considered. As a consequence, an uncertainty assessment model for measuring internal geometries by CT is currently not available.

Therefore, this work proposes an alternative method for those specific cases in which a previous calibration by a more accurate coordinate measuring system or CMS is unfeasible (due to inner parts, 3D complex geometries, etc.). The method is based on the estimation of the maximum permissible error (MPE) of the micro-CT system. This is achieved by the MPE experimental determination using several calibrated reference artefacts. The MPE value considers the influence of different geometries, sizes, positions, and orientations, as well as the reproducibility of operation conditions, data processing, and the effect of environmental conditions. To validate the proposed method, a micro injection molded workpiece used for micro mechanical material testing that can be characterized by both micro-CT and CMS is employed. The uncertainty evaluation of its measurands are estimated using two approaches: first, according to a novel proposed method; and secondly, according to the most accepted standard [13]. Furthermore, and considering the high number of influence factors in CT [14], such as data processing, two different techniques are used for the surface extraction to perform the measurements: one method based on the local threshold method [15]; and another method based on the 3D Canny algorithm [16]. Once confirmed that the new approach is suitable, the measurement and uncertainty evaluation of a miniaturized dental file with 3D complex geometry is carried out. The estimated measurement uncertainties obtained are eventually compared with the component’s calibration and tolerances to validate the measuring capability of the micro-CT system and its calibration procedure.

## 2. Measurement Uncertainty Assessment Using CT: A Review 

In order to justify the necessity of an alternative solution for the accuracy evaluation of CT dimensional measurements, a short review is presented here. There are different methods to achieve traceability by using coordinate measuring systems or CMSs (i.e., tactile and optical). However, due to the high number of influence factors in CT measurements (hardware, data processing, workpiece, environment, operator, etc.) the existing standards for CMSs cannot be directly applied to CT systems. Despite these differences, initial CT uncertainty results were estimated based on these international guidelines, as can be found in the literature and will be described later in this section. Only the national VDI/VDE 2630 Part 2.1 [13] was specifically developed for CT metrology systems. However, the VDI/VDE 2630 Part 2.1 [13] standard has as a main limitation the fact that it only considers the uncertainty assessment of measurements on external geometries and not of internal geometries.

Three main different applied procedures to estimate the measurement uncertainty exist and are classified as follows [9,17,18]:

Model-based uncertainty budgets, such as the Guide to the Expression of Uncertainty in Measurement (GUM) method [19] and the one described in the International Organization for Standardization (ISO) standard 14253-2 [20]. Model-based uncertainty budgets require a model equation and apply the error propagation theory. For CT systems, the disadvantages of applying model-based uncertainty budgets are: the complexity of the measurement process, the numerous factors of influence, their complex quantification and interaction, and their variability over time (i.e., drift). Therefore, there is a consensus in the scientific research community: analytical approaches to calculate the measurement uncertainty of CT suppose a complex and extensive work, which seems to be extremely difficult to carry out reliably and not appropriate to be applied in an industrial environment. Only simplified studies have been conducted so far considering the GUM approach [21,22]. On the other hand, the iterative and simplified method according to ISO 14253-2 has received attention for its application in CT measurements [23,24].

Simulation methods (e.g., Monte-Carlo simulation [25]) employed for uncertainty estimation should be based on a complete characterization of the measurement process. Factors not considered in the model should then also be estimated by other means (e.g., experimentally). In addition, the simulated results have to be validated experimentally. Due to the complexity of the CT measurements, only very few attempts to calculate the measurement uncertainty from simulations are reported [26,27]. In these studies, the comparison of measured and simulated CT data is achieved by developing a specific software tool for simulation of the CT image acquisition process.

Empirical methods according to ISO 15530-3 [28] and the specific standard for CT VDI/VDE 2630 Part 2.1 [13] are based on the assessment of the measurement uncertainty by means of a calibrated workpiece. Despite the high number of repeated experiments necessary for statistical validation, this procedure is currently regarded by many authors as the most accepted for CT measurements. Several articles consider and apply the ISO 15530-3 standard [17,18,29,30] and the VDI/VDE 2630-2.1 version [31]. However, the VDI standard in its current version only considers geometries accessible from outside the workpiece, not considering the measurement characterization capability of internal features that is the most distinguishing advantage of CT and µCT metrological systems. In addition, when applying these standards, outer geometries are required due to the necessity of a previous calibration of the workpiece, normally carried out by CMSs, using the same strategy and conditions for the part to be inspected.

Other procedures are combinations of the mentioned methods and a consequence of expert knowledge. Due to the lack of international standards for the uncertainty assessment of measuring systems with CT sensors, and the limitation of the specific CT guideline VDI/VDE 2630-2.1 only to external geometries, it is concluded that a new approach for measurement uncertainty evaluation using CT is needed. Consequently, the present work aims at proposing and validating an alternative method for these specific cases when a previous characterization with a more accurate measurement system of the analyzed workpiece is not possible (due to inner parts, 3D complex geometries, etc.). The proposed method described in this research is based on the estimation of the maximum permissible error (MPE) of the micro-CT system. The MPE was experimentally determined by using several calibrated reference artefacts with different geometries (spheres, internal and external geometries, different materials, etc.). This term, according to the International Vocabulary of Metrology (VIM) [32], represents the extreme value of an error permitted by specifications for a given measuring system, between its indicated value and the corresponding true value. By using this MPE, an adaptation of the ISO 14253-2 is developed for measuring uncertainty assessment using CT.

## 3. Uncertainty Assessment Based on the Experimental MPE of the CT System 

The aim of this work is to study and validate a new approach of measurement uncertainty evaluation for computed tomography. This method is based on the determination of a reference maximum permissible error (MPE), which is experimentally estimated by using several reference items. To achieve traceability, these artefacts are previously calibrated by a CMS and then measured by the micro-CT system to determine its representative MPE. To validate the proposed approach, the specific standard developed for CT systems is also applied. The uncertainty of a micro three-dimensional component is evaluated, first, considering the proposed alternative analysis and, secondly, according to the mentioned standard VDI/VDE 2630 Part 2.1 (June 2015) [13].

**Figure 1:**
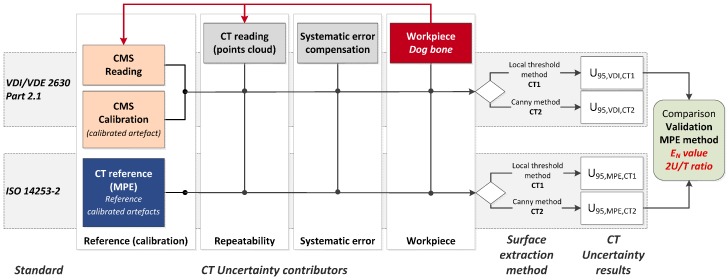
Uncertainty assessment procedures using computed tomography: validation scheme of the proposed method.

The workflow of the procedure validation is shown in Figure 1. Two approaches are applied to assess the measurement uncertainty of a particular workpiece (named dog bone): one based on the estimated MPE and an adaptation of the ISO 14253-2, and another one according to the VDI/VDE 2630-2.1. The error sources to be considered in both uncertainty budgets are those included in the VDI/VDE 2630-2.1 standard, which is focused on CT sensors. Considering k = 2 (coverage factor for a confidence interval of 95.45%), the expression of the uncertainty is given by Equations (1) and (2), respectively. The difference between both approaches is the reference value assumed for the CT system (u_ref_ and u_cal_).

(1) $$U_{95,\mathrm{MPE},\mathrm{CTi}}=k\sqrt{u_{\mathrm{ref}}^{2}+u_{p}^{2}+u_{w}^{2}+u_{b}^{2}}$$

(2) $$U_{95,\mathrm{VDI},\mathrm{CTi}}=k\sqrt{u_{\mathrm{cal}}^{2}+u_{p}^{2}+u_{w}^{2}+u_{b}^{2}}$$

where: u_cal_ and u_ref_ represent the standard uncertainty of calibration; u_p_ is the standard uncertainty of the measurement procedure (repeatability); u_w_ is the standard uncertainty of the material and manufacturing variations of the measured process; u_b_ is the standard uncertainty associated with the systematic error of the measurement process. The i-index (i = 1, 2) refers to the two surface extraction methods described below: CT1 (local threshold method) and CT2 (Canny algorithm), respectively.

As summarized in Table 1, the three last terms are common for both methods. The difference lies in the first term that refers to the calibration process. On the one hand, the term u_ref_ is experimentally determined by using several calibrated reference artefacts. These different artefacts and their measurement procedure by computed tomography have to be considered acceptable to define the global MPE of the CT system. Since error sources are known, the standard ISO 14253-2 [20] can be used to estimate measurement uncertainty. On the other hand, the VDI/VDE 2630-2.1 standard is based on the task-specific calibration, where a calibrated workpiece is used. Therefore, the term u_cal_ is the standard uncertainty due to the calibration of the workpiece (usually by a CMS). The factor u_drift_ is not computed in this work as scheduled recalibration dates are not considered at this point. The estimated uncertainties are eventually compared with the component’s calibration and tolerances to validate the measuring capability of the micro-CT system and its calibration procedure. Hence, the E_N_ value and the 2U/T ratio were calculated for all measurands (see Figure 1).

To evaluate the influence of the processing, two different techniques are applied for the surface extraction to perform the measurements: CT1 or the local threshold method [15] and CT2 based on the 3D Canny algorithm [16]. The CT1 method is a well-known technique based on the determination of a threshold value to distinguish between two materials; i.e., based on a similarity principle. Points darker than the threshold value are considered one material (e.g., air) and the brighter ones are considered the other one (e.g., part). After that, a 3D grey value interpolation that takes into account the surrounding volume is performed in order to improve the accuracy. To estimate the threshold value, the ISO50 method is widely used, but it is demonstrated that it can offer inaccurate results [33]. Hence, in this work, this value has been corrected in order to find another one which minimizes the deviation between a reference value of one measurand (obtained by an additional and more accurate measurement process) and its measured value. On the other hand, the CT2 method, previously developed by the authors [16], is based on a gradient algorithm (different from the CT1 method) and is an adaptation of the 2D Canny algorithm to the 3D case of this problem. As explained in [16], its implementation needs four steps: first, a preliminary surface detection based on the image gray values; second, a sub-voxel resolution refinement by a 3D interpolation; third, the measurement process itself; and fourth, a correction based on the same reference value as CT1.

Each of the two surface extraction techniques shows different advantages and drawbacks when measuring simple geometrical features. Local threshold techniques are widely used in commercial CT systems nowadays and provide accurate results regarding the bias error. However, the main disadvantage of these methods, based on the similarity principle, is that they are time-consuming and, in many cases, they need reference values for most of the elements to be measured, showing some problems of clearly determining the corresponding edges. On the other hand, the edge detection technique based on the Canny algorithm provides an edge location with improved capability. This is an advantage when measuring 3D complex geometries and even multi-material parts. This method significantly reduces the probability of losing the real edges of the workpiece and of detecting false edges due to image noise. This is the determinant for an improved distinction and determination of the edges when they are in contact with either air or a material different from the base material, i.e., fixture. In addition, the measurement uncertainty values obtained are often lower than that with the thresholding technique [16]. Nevertheless, the systematic error of this technique, based on discontinuity, is usually higher than with the local threshold method, and, as mentioned before, it still needs at least one reference dimension in order to compensate and eventually obtain accurate results. The application of both surface extraction methods in this research allows the study of their respective behavior when used for complex geometry measurements.

### 3.1. Uncertainty Assessment Based on the Experimental MPE of the CT System 

#### 3.1.1. Standard Uncertainty of the CT System Used as a Reference (u_ref_)

The first uncertainty influence factor of the solution presented in this work is u_ref_. The micro-CT system MPE was experimentally determined by using several calibrated reference artefacts with maximum calibration uncertainties lower than ±3.0 µm for all the dimensions used:

1. Item 1, called “CT tetrahedron” of the CT Audit international intercomparison [34]. It consists of four calibrated spheres made of synthetic ruby mono-crystal supported by a carbon fiber frame.
2. Item 2, called “Pan Flute Gauge” (CT Audit), which consists of five calibrated tubes made of borosilicate glass supported by a carbon fiber frame [34].
3. The Calotte cube that was developed by the Physikalisch-Technische Bundsanstalt (PTB) (CT Audit) and consists of 75 spherical calottes on three sides of a titanium hollow cube [34].
4. A 42 mm-long replica step gauge made of bisacryl material for dental applications [35].
5. A commercial Lego^®^ brick with eight cylindrical features (i.e., knobs) on the top side that was made of acrylonitrile–butadiene–styrene (ABS), which is an engineering thermoplastic polymer with good dimensional stability.

These different artefacts and their measurement procedure have been considered acceptable to define the global MPE of the CT system, since all of the following premises ensure the generality of the results obtained for the MPE of the measurement µCT system:

▯ All the reference artefacts have been previously calibrated by a CMS, following a similar measurement procedure, so that the MPE is determined by evaluating the deviations between the µCT and the CMS measurements for the same measured volume. Considering the different sizes of the artefacts, the dimensional range covered is up to 40 mm in length in all directions.
▯ Different geometries have been characterized: spheres, internal and external (e.g., inner and outer diameters), geometries both threshold-dependent and independent, different types of patterns (e.g., crenellated and grooved forms), etc.
▯ Despite the multi-material nature of the artefacts assembly, the measured parameters are from homogeneous parts. The different X-ray absorption is evaluated by the measurement of different reference materials, and the function of the electron density of the object.
▯ The µCT measurement has been fulfilled in different positions and orientations of the workpieces. In each position, several repetitions have been completed.
▯ According to the magnification, the voxel size of the µCT measurements, used for the calibration with the reference artefacts and for the characterization of the workpieces, is comparable. The number of projections or the angle increment between the radiographs (in a 360° scan) that define the point density is also equivalent.
▯ Data processing procedures, such as correction techniques and filtering, reconstruction algorithms and fitting procedures, have been identical for all CT measurements. Considering the surface detection technique, this work analyses two methods that result in two different MPE values: one for CT1 (local threshold method) and another one for CT2 (Canny algorithm). Regarding the uncertainty calculation, this is the only difference between the respective data processing procedures.
▯ The exposure time and the environmental conditions (temperature, humidity, vibrations, etc.) have been controlled to assure reproducible measurements by the CT.

**Figure 2:**
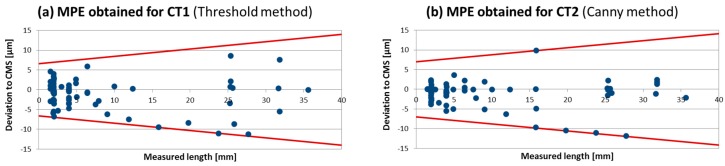
Measured length vs. Deviation to the CMS reference. (**a**) MPE for the CT1 or local threshold method; (**b**) MPE for the CT2 or 3D Canny method.

Once the deviations between the micro-CT and CMS measurements along the considered volume are determined, the MPE expression as a function of the measured length can be estimated. As mentioned before, two different techniques were applied for the surface extraction to perform the measurements. The MPE obtained for CT1 (local threshold) and CT2 (3D Canny) were respectively: MPE_CT1_ = 6.6 µm + (L/5.4) μm and MPE_CT2_ = 7.0 µm + (L/5.6) μm, where L is in mm. These values are considered for a measurement range up to 40 mm and considered sufficiently conservative for the applied case (see Figure 2). Thus, the term characterized by the MPE of the CT is calculated as follows (Gaussian distribution):

(3) $$u_{\mathrm{ref}}=\frac{{\mathrm{MPE}}_{\mathrm{CTi}}}{2}$$

#### 3.1.2. Standard Uncertainty Due to the Calibration of the Workpiece (u_cal_)

The first uncertainty influence factor according to the VDI standard is u_cal_. The term represents the uncertainty of measurement due to the calibration uncertainty of the workpiece by a CMS (tactile or optical), and it is calculated as follows:

(4) $$u_{\mathrm{cal}}=\frac{U_{\mathrm{cal},\mathrm{CMS}}}{k}$$

In this work, the CMS employed was an optical coordinate measuring machine (OCMM) DeMeet 220 using diascopic illumination with a light ring, a lens with 2× magnification, and a field of view of 3111 µm × 2327 µm. The uncertainty assessment of the OCMM measurements considers three influence factors: MPE of the OCMM from the machine calibration; measurement repeatability; and the influence of the temperature. The OCMM uncertainty for length measurements in the 100–1000 μm range was evaluated, resulting in the maximum permissible error MPE_OCMM_ = 1.7 μm. For the measurements with a length L > 1 mm, the maximum permissible error of the OCMM obtained is: MPE_OCMM_ = 5 μm + (L/150) μm (L in mm). Regarding environmental conditions, the influence of the temperature has also been included in the uncertainty budget. The OCMM is placed in a metrology laboratory with standard conditions of temperature 20 ± 1 °C and humidity 50–70% controlled 24/7.

#### 3.1.3. Standard Uncertainty of the Measurement Procedure (Repeatability)

According to the VDI standard, this term represents the standard measurement uncertainty contributor due to the measurement process, i.e., the standard deviation of the repeated measurements. It is recommended to perform the measurement 20 times under varying conditions (e.g., stability of the X-ray tube parameters, mounting and remounting of the workpiece, different operator, etc.). In this case, we consider up to 10 repetitions (n = 10) of each measurand, but from four workpieces (m = 4) previously calibrated by a CMS (in this case an OCMM). The use of four different items modifies the estimation of this term (see Equations (5) and (6)), because several standard deviations have been considered. This estimation reflects the effects of material composition and shape, so that these factors need not to be estimated separately in u_w_ (that includes the error factors associated to the workpiece).

(5) $$u_{p,j}=\sqrt{\frac{1}{n-1}\sum_{i=1}^{n}{\left(y_{i}-\bar{y}\right)}^{2}}$$

(6) $$u_{p}=\sqrt{\frac{1}{m}\sum_{j=1}^{m}u_{p,j}^{2}}$$

#### 3.1.4. Standard Uncertainty from the Material and Manufacturing Variations

The term u_w_ is associated with two uncertainty sources as shown in Equation (7): u_w1_ represents the variations in the mechanical properties of the workpiece; and u_w2_ considers the variations in the CTEs (coefficient of thermal expansion) of the workpiece. The first factor has been previously included in u_p_ (effects of material composition and shape). For the second one, a minimum uncertainty of 20% of the value shall be assumed [13]. This recommendation from the VDI/VDE 2630-2.1 is due to the fact that the standard uncertainty of the coefficient of thermal expansion is often unknown. In this work, a rectangular statistical distribution and a CTE variation of 20% has been established for this term.

(7) $$u_{w}=\sqrt{u_{w1}^{2}+u_{w2}^{2}}$$

#### 3.1.5. Standard Uncertainty Associated with the Systematic Error

The term u_b_ considers the influence of two effects as shown in Equation (8): u_b1_ evaluates the influence of the temperature variation during the µCT measuring process (ΔT = ±2 °C); and u_b2_ estimates the systematic error that is related to the surface detection techniques: local threshold and 3D Canny methods, or CT1 and CT2, respectively.

(8) $$u_{b}=\sqrt{u_{b1}^{2}+u_{b2}^{2}}$$

The second contribution depends on the measurement correction in order to first calculate, and then compensate for the bias error, so that the standard uncertainty of the scale factor and the applied offset determination are here considered.

### 3.2. Validation of the Proposed Alternative Analysis: Dog Bone Case Study 

#### 3.2.1. Dog Bone Workpiece

**Figure 3:**
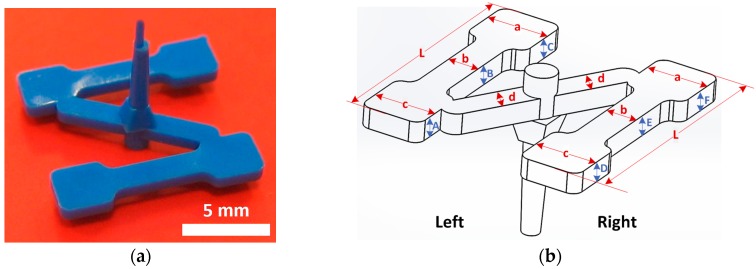
Dog bone. (**a**) Micro injection molded component. (**b**) Characteristic dimensions: left and right lengths (L, a, b, c, d) and thicknesses (A, B, C, D, E, F).

The part used to evaluate the proposed methodology for uncertainty assessment in CT is a miniaturized dog bone (Figure 3a). This specimen is made of acetal polyoxymethylene (POM) copolymer and used for micro mechanical material testing. In this case, five dimensions of four items (DB1, DB2, DB3, and DB4) were verified at both the left and the right side of the part: lengths (L, a, b, c, d) and thicknesses (A, B, C, D, E, F), respectively (Figure 3b). Nominal dimensions are: length L = 11.80 mm; lengths a,c = 3.00 mm; length b = 1.50 mm; length d = 1.35 mm; and thicknesses A to F = 1.00 mm. The measurement protocol to verify the dimensions is detailed in [34]. Table 2 summarizes all the parameters of the dog bone, their description, nominal values, and tolerances.

#### 3.2.2. CT Measurement

A General Electric eXplore Locus SP cone-beam micro-CT machine was used for the CT measurements. Its X-ray source voltage range is between 50 and 90 kV, the maximum resolution or minimum voxel size of 8 µm and a cylindrical working volume of 44 mm in diameter and 56 mm in height. During scanning the temperature was recorded inside the machine, obtaining a temperature range of 20 ± 2 °C. The scanning parameters used are presented in Table 3. These parameters were obtained by the experimented operator after an iterative process in order to provide a high quality image.

The dog bone geometry (Figure 3) does not allow for the application of the correction procedure described in [16], due to the lack of correlated measurands. For this reason, the trend of the errors has been used to correct the results, assuming that the relation between the measured length and the obtained deviation from the reference follows a linear regression [15]. The slope of the regression line is the scale factor and the y-intercept is the offset correction as demonstrated in [15].

#### 3.2.3. Uncertainty Results (Dog Bone): Analysis and Discussion

**Figure 4:**
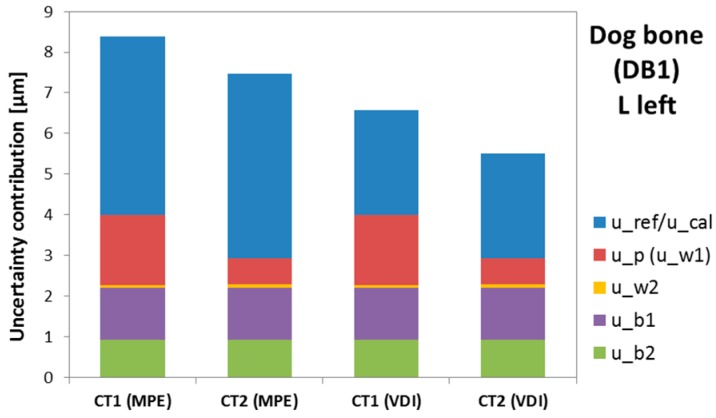
Dog bone (DB1): Uncertainty contributors obtained by the micro-CT system with two approaches (MPE and VDI) and two surface extraction techniques used (CT1 and CT2) for L left.

The uncertainty results by applying the studied solution based on the MPE of the µCT system and by considering the VDI/VDE 2630-2.1 are presented here. In Table 4 and Table 5 the uncertainty contributors and expanded uncertainty (U_95_, k = 2) obtained by both approaches are shown, respectively. Due to the almost equivalent results of the four specimens, only the dog bone DB1 results are included. Four measurands have been selected as representative from the entire workpiece: L left, a left, b right, and E thickness (see Figure 3). In addition, the results from the two surface extraction techniques CT1 and CT2 are also compared. In Figure 4 the difference between the uncertainty contributors can be observed, illustrated by the results obtained for the L left parameter of DB1.

The terms u_ref_ and u_cal_ were found to be the most relevant. The second major uncertainty contributor depends on the surface extraction technique. Differences between the local threshold method and 3D Canny algorithm were shown in the u_p_ term. Since the 3D Canny adapted method provides lower deviations and needs an easier correction than the local threshold, that technique results in a lower maximum expanded uncertainty. Despite the different surface extraction techniques, u_b1_ and u_b2_ (standard uncertainty associated with the systematic error) are similar for CT1 and CT2, and both suppose 25–40% of the total contribution. The uncertainty contribution resulting from the workpiece material due to environmental condition variations (u_w2_) had a very low influence (lower than 0.1 μm) on the final combined uncertainty. With regard to the differences between uncertainty assessment procedures, the standard VDI/VDE 2630-2.1 concludes in a more precise result (u_cal_ < u_ref_). Nevertheless, for those cases where the task-specific calibration is unfeasible, the MPE estimation of the CT system can be an adequate solution, as demonstrated by the E_N_ value analysis and the tolerance verification capability discussed in the next sections.

##### E_N_ Value Analysis

To validate the expanded uncertainty results in relation to the measuring uncertainty of the used instruments, CMS (OCMM in this case), and micro-CT system, the E_N_ value was calculated for all measurands. The E_N_ value represents the deviation between a measured value (i.e., obtained by the µCT system in the present case) and the corresponding calibrated value (i.e., obtained by the CMS) in relation to their respective stated uncertainties [36]. It is given by Equation (9):

(9) $$E_{N}=\frac{\left|(CT_{\mathrm{meas}.\mathrm{value}})-({\mathrm{CMS}}_{\mathrm{ref}.\mathrm{value}})\right|}{\sqrt{U_{\mathrm{CT}}^{2}+U_{\mathrm{CMS}}^{2}}}$$

Then, if E_N_ < 1, there is a satisfactory agreement between the two values, otherwise there is no agreement among them. Clearly, a large stated uncertainty of the measurements by the CT system would lead to a small E_N_, suggesting that its measuring uncertainty might be overestimated. The E_N_ value was calculated for all CT measurements in relation to their corresponding OCMM measurements. The results for all the measurands with E_N_ < 1 percentages are summarized in Table 6. The analysis shows that a relatively high number of measurements are characterized by an E_N_ value below 1. Furthermore, measuring results are substantially influenced by the employed surface extraction technique. The CT2 or 3D Canny surface extraction technique provided results with higher agreement with the reference values than the CT1 technique (i.e., based on local thresholds). On the other hand, the measurement of the a_left_ and c_right_ appear to be particularly challenging, with 25% or less of the measurements characterized by E_N_ < 1. The reason for this result is the position of this measurand in the cantilever zone of the workpiece (see Figure 3b).

##### Tolerance Verification Capability (2U/T Ratio)

**Figure 5:**
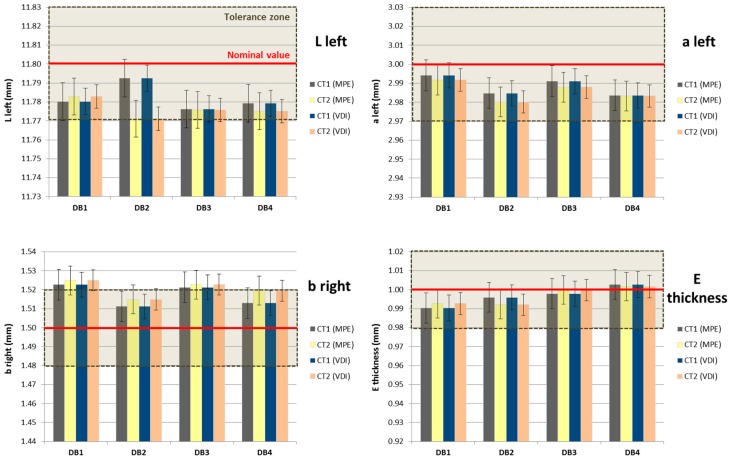
Dog bone: Measurement results of the four workpieces and selected measurands obtained with both calibration procedures (MPE and VDI) and using two surface extraction techniques (CT1, CT2). L left (up and left); a left (up and right); b right (down and left); E thickness (down and right).

The tolerance verification capability analysis is performed to verify whether the measured components (micro injection molded dog bones) are within the specifications and whether the CT system has a sufficiently low measuring uncertainty so that it is suitable for tolerance verification in micro manufacturing. This is demonstrated by evaluating the 2U/T ratio (where U = expanded uncertainty of the CT measurement and T = tolerance of a certain measurand as specified in the component design). Considering the four measurands: L left, a left, b right and E thickness (see uncertainty results presented in Table 4 and Table 5), the relations between the nominal value, tolerance limits, measured value, and uncertainty range are presented in Figure 5. It can be observed that the results in terms of tolerance compliance are similar when using either the µCT system MPE approach or the uncertainty assessment according to VDI/VDE 2630-2.1, so both methods give comparable results. Furthermore, the differences between CT1 and CT2 are shown as well.

The analysis of the CT measuring capability is carried out by evaluating the ratio between the uncertainty (2U) and the tolerance zone (T) for all dimensions of the dog bone. For effective tolerance verification the 2U/T ratio must be lower than 20% but, when the tolerances are tight, as in the case of micro-manufactured products, an enlargement of this ratio up to 40% is allowed [13,37]. In this case, all the measurands meet with this rule. In other words, 100% of the characterized dimensions fulfill the requisite of 2U/T ≤ 0.4, for both the procedure based on the assessment of the measurement uncertainty by means of the calibrated workpiece and for the proposed alternative solution with the MPE of the µCT system. Furthermore, the presented method shows a ratio closer to this superior limit in a high number of measurands for both CT1 and CT2 surface extraction techniques.

##### Proposed Uncertainty Assessment Method: Conclusion

The conclusions that can be drawn from these results are here summarized, considering the dog bone case study used to validate the proposed alternative of uncertainty assessment by using CT. In terms of uncertainty, the results obtained by both approaches (MPE and VDI) are similar, although the standard VDI/VDE 2630-2.1 concludes in a more accurate result due to the fact that u_cal_ < u_ref_. Nevertheless, the maximum permissible error has been overestimated (conservative approach), considering different materials, sizes, and geometries. With the obtained uncertainty results, a relatively high number of measurements are characterized by an E_N_ value lower than 1. In addition, the tolerance compliance is similar either using the MPE of the CT system or the uncertainty assessment according to the VDI/VDE 2630-2.1. All the measurands meet with the rule of 2U/T ≤ 0.4. However, the alternative defined in this work results in ratios nearest to the upper limit. In short, both methods give comparable results. Even in the case of an overestimated MPE of the micro-CT system, the presented alternative analysis is feasible for several specific cases. These findings clearly show that the novel proposed method can be employed to calculate the uncertainty of a CT system. The method is particularly useful for all those cases in which it is not possible to perform the calibration of the workpiece with other measurement techniques (for example, in the case of the presence of measurands on inner geometries, 3D complex geometries, etc.). 

## 4. Uncertainty Assessment of a 3D Complex Micro Component

The main aim of this work is to propose and validate a specific method for uncertainty assessment using micro-computed tomography. This necessity appears in cases when a task-specific calibration cannot be carried out. In order to further validate the method, it was applied to a miniaturized component for medical applications, a dental file, that has a 3D complex geometry and, for which, a calibration by either a tactile or optical CMS is not available. 

**Figure 6:**
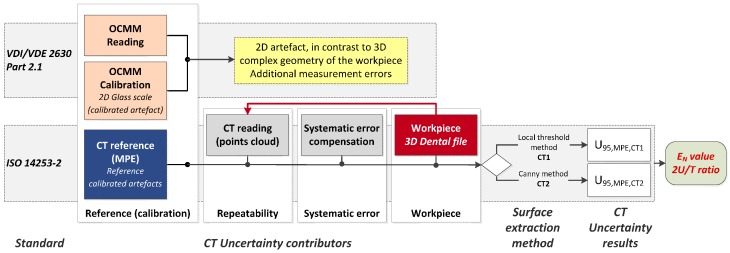
Uncertainty assessment procedure using computed tomography: characterization of a workpiece having a 3D complex geometry.

The procedure workflow applied in this section is represented in Figure 6. First, a dental file with variable diameters and helix angles is measured by a µCT system. The dental file has been preliminarily measured by the OCMM employed for the dog bone. The optical system has been verified with a two-dimensional flat mask calibration artefact, in contrast to the measurands in the dental file that are clearly of a three-dimensional nature. This discrepancy may lead to additional measurement errors by the OCMM. Thus, the CT characterization becomes a suitable alternative solution for three-dimensional micro metrology tasks, when CMS based on optical and tactile techniques present this kind of limitation; i.e., the calibration of true 3D measurements cannot be achieved. The proposed method deals with the fact that currently available standards for uncertainty assessment are not adequate in these specific cases; i.e., the measurement of complex 3D geometries.

### 4.1. Application of the Method: Dental File Case Study 

#### 4.1.1. Dental File Workpiece

**Figure 7:**
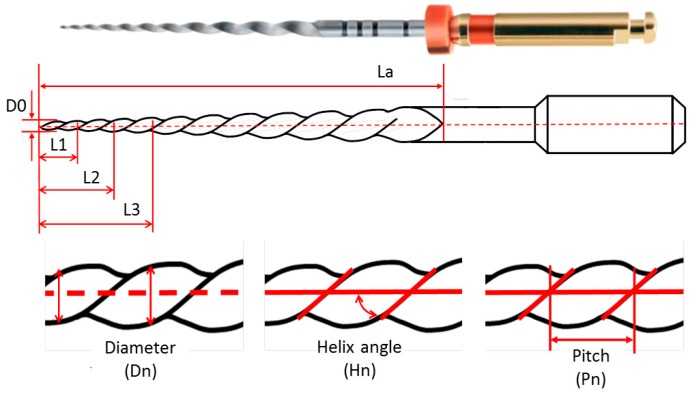
Dental file and characteristic dimensions: length of the active cutting part (La); diameter (Dn); helix angle (Hn); pitch (Pn).

A ProTaper F2 finishing file (produced by Dentsply Maillefer, York, PA, USA) is used for the study [38,39]. This instrument is manufactured in a Ni-Ti alloy. Its complex 3D geometry is defined by different dimensions of such root-canal instruments include lengths, diameters, helix angles, and pitches, according to the standard ISO 3630-1:2008 [40]. The dimensions to be verified in the working part are the following (see Figure 7): (i) length of the active cutting part (La); (ii) variable diameter along the file length (Dn, n = 0, 1, 2, …, 13), with D0 as the diameter at the file tip and D1, D2, D3, etc., as the diameters at 1, 2, 3, etc., mm along the file axis, respectively; (iii) helix angle (Hn, n = 1, …, 10) or the angle formed between the helix and the file axial axis, with the first one (H1) being the angle formed between the tip and the base of the file; (iv) helix pitch (Pn, n = 1, …, 11) or the distance between a point in the forward edge and its corresponding point in the adjacent edge along the file longitudinal axis, with P1 being the first helix pitch starting from the tip of the file.

The nominal dimensional values available for this dental file are, according to [38]: a cutting segment (La) of 16 mm in length, a tip diameter (D0) of 0.25 mm, a fixed conicity of 8% between D0 and D3, and a variable conicity from D3 to D13 along its axis. The ISO 3630-1:2008 [40] provides guidelines to specify tolerances for diameters and lengths. On the contrary, guidelines for the tolerances of helix angles and helix pitches are not specified, neither by the standards, nor by the manufacturer. Therefore, the tolerance values used in this study were limited to those available in the standard. Table 7 summarizes all the parameters of the dental file, their brief description, nominal values, and tolerances.

#### 4.1.2. CT Measurement

**Figure 8:**
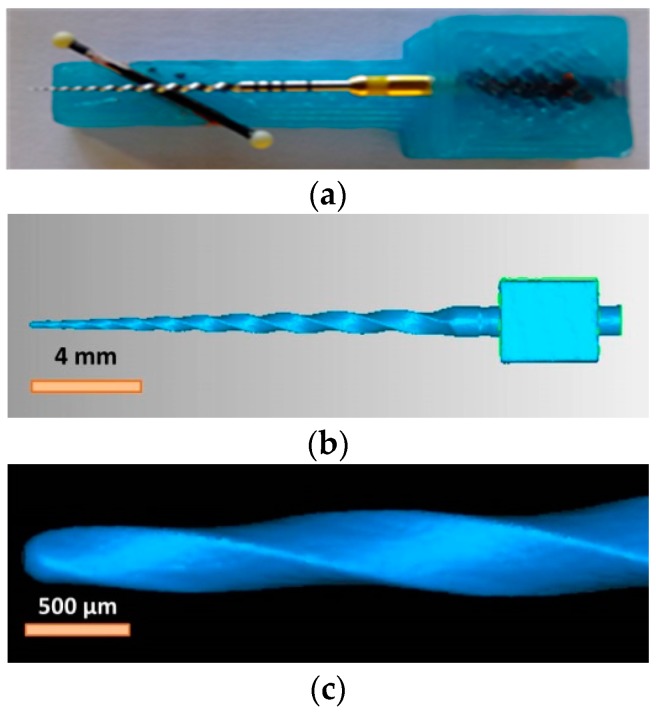
(**a**) Dental file and miniaturized ball bar during the measurement on the CT scanner; (**b**) 3D volume reconstructed from the CT scan of the complete dental file; (**c**) 3D volume reconstructed from the CT scan of the dental file tip and of the helix geometry.

The file was measured in the micro-CT system together with the miniaturized ball-bar reference standard previously calibrated (Figure 8a), in order to determine the scale factor and correct the measurements obtained after scanning. In particular, the ball bar allowed the determination of the scale factor independently of the threshold strategy because it is based on the measurement of the distance between the centers of the two spheres [9,23,27]. A cube is attached to the workpiece at the bottom of the cutting area of the file, in order to use their faces as reference planes (Figure 8b). The dental file measurements were performed four times, each one determined by an orientation of the face of the cube resting parallel to the rotary table. During scanning, the parameters used were those included in Table 8. The temperature recorded was in the range of 20 ± 2 °C.

An example of the 3D volume reconstructed after the image analysis process can be observed in the complete scan of the dental file (including the reference cube used for the alignment of the measurement) in Figure 8b, and a detail of the dental file tip and of the 3D complex helix geometry in Figure 8c. The procedure of reconstruction and the surface extraction method were the same as in the dog bone case. However, the systematic error contribution is calculated differently. The ball bar allowed the determination of a precise global scale factor independently of the threshold strategy, because it is based on the measurements of the distance between the centers of the two spheres. A global compensation is applied here, since the pixel size of the X-ray detector is periodically calibrated and it is compensated by the reconstruction software. Hence, the first error contribution is due to the adjustment of the scale factor. Alternatively, in other research works, single correction factors or a mean correction factor are calculated separately to rescale the voxel size and compensate for random effects [29]. The second uncertainty influence is due to the deviations when extracting the surfaces boundary. This term evaluates the standard uncertainty of the variable errors by locally adapting the ISO factor (CT1) and offsets due to bias compensation (CT2) obtained, respectively.

#### 4.1.3. Uncertainty Results (Dental File): Analysis and Discussion

The results of the procedure for uncertainty assessment of the measurements of the dental file proposed in this work based on the MPE of the micro-CT system are described in this section. The final expression of the uncertainty to obtain U_95,MPE,CT1_ and U_95,MPE,CT2_ (coverage factor k = 2) is given by Equation (1). In Table 9, the uncertainty contributors and maximum expanded uncertainty (U95, k = 2) obtained by both CT1 and CT2 are shown for some selected dimensions of the dental file (e.g., D11, La, P6, H6). The angle measurement uncertainty was estimated by applying the error propagation law, as described in the GUM [19].

As can be observed, the U_95,MPE,CT1_ and U_95,MPE,CT2_ values for lengths mainly depend on the u_ref_, which is conservatively estimated from the MPE of the micro-CT system. The u_p_ term was found again to be one of the most relevant, together with the u_ref_. The contribution of the systematic error is certainly different (u_b_), in view of the surface extraction method applied. Generally, the 3D Canny algorithm leads to more accurate results. The u_w_ contribution had a very low influence in the final combined uncertainty. To validate these results, the E_N_ value and the 2U/T ratio are analyzed below.

##### E_N_ Value Analysis

As previously done for the dog bone case, the E_N_ value was calculated for all measurands of the dental file. In Table 10, the percentage of dimensions with E_N_ < 1 is shown. Generally, the 3D Canny surface extraction technique provided results with higher agreement with the reference values. This is due mainly to lower deviations from the reference values, indicating that its use is advantageous as compared to the threshold technique. On the other hand, the measurement of the active cutting part length and of the helix angle appear to be particularly challenging, with 50% or less of the measurements characterized by E_N_ < 1. This behavior is caused by a particularly difficult definition of both measurands, because they are primarily based on 3D geometrical features, that are, as far as the length La is concerned, the determination of the starting point at the bottom of the dental file and, for the helix angle, the determination of the tangent on the cutting edge.

##### Tolerance Verification Capability (2U/T Ratio)

**Figure 9:**
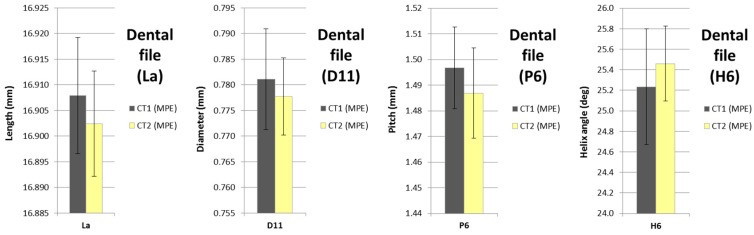
Dental file: Measurement results of the four workpieces and selected measurands (La, D11, P6, H6) using two surface extraction techniques (CT1, CT2).

**Figure 10:**
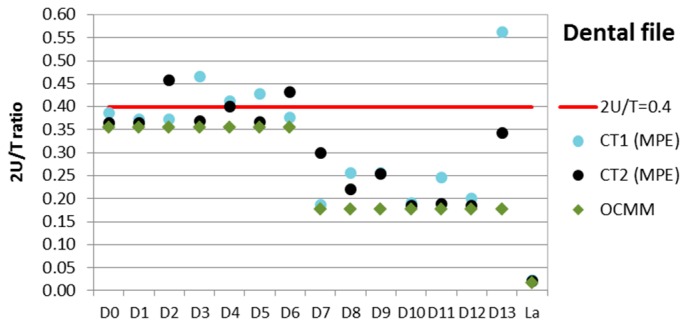
Dental file: 2U/T ratio calculated for all CT measurements with tolerance specification (La and variable diameter), obtained with the proposed calibration alternative (MPE) using two surface extraction techniques (CT1, CT2).

Tolerance specifications are only defined for the length of the active cutting part (La) and the variable diameters (D0 to D13). Nevertheless, in Figure 9 the measurement results of the four selected measurands (La, D11, P6, H6) are shown, including uncertainty ranges. Additionally, the results of the 2U/T ratio for the diameters and the active length (La) are summarized in Table 11. For the active length, with a wider tolerance, all the three techniques meet the requirement. It can be observed that the ratio obtained with the OCMM for the diameters and the active length is always smaller than 0.4 (see also Figure 10). In the case of the CT1 and CT2 measurements, for the dimensions from D0 to D6, where the tolerances are smaller, the percentage number of measurements with a ratio 2U/T ≤ 0.4 is slightly above 57% and 71%, respectively. In contrast, for dimensions from D7 to D13, where the tolerances are larger, the relationship 2U/T ≤ 0.4 is achieved by around 85% dimensions with CT1 and 100% dimensions with CT2. Therefore, despite higher uncertainties and challenges in performing CT scanning metrology, its applicability towards tolerance verification on complex geometries appears promising.

## 5. Conclusions

Micro-computed tomography still presents limitations in terms of the accuracy and precision of measurements. However, it appears that its measuring capability is approaching the requirements for effective tolerance verification of high precision miniaturized components. The challenging issues for the complete acceptance of µCT for metrology purposes are the limitations directly related to the measurement uncertainty evaluation. In the present work, an alternative method is proposed to provide traceability to the 3D measurements obtained by a micro-CT system for the verification of dimensions on miniaturized components. With the use of this method, the specific cases of characterization of 3D complex geometries, inner parts, etc., can be fulfilled. This is particularly convenient in all those cases in which a previous calibration of the analyzed workpiece with a more precise measurement system is either not available or it is simply not possible, as the measuring task is beyond the measuring capability of existing optical or tactile CMS. The proposed method is based on the estimation of the maximum permissible error (MPE) of the CT system. The MPE is experimentally determined by using several calibrated reference artefacts with different geometries, sizes, positions, and orientations, for which the calibration values are easily determined and are available, in reproducible procedure conditions. Therefore, this MPE may be considered as a global expression of the error along the dimensional range calibrated. In brief, this article proposes an alternative to uncertainty assessment by using CT. Its main advantage is that a previous calibration of a similar component by a more accurate coordinate measuring system (CMS) is not needed. This overcomes the typical limitations of optical and tactile techniques, particularly when measuring miniaturized components with complex 3D geometries and their inability to measure inner parts. The method validation has been carried out by comparison with the most accepted procedure based on the assessment of the measurement uncertainty by means of the calibrated workpiece. The results presented in this work demonstrate that the proposed new method can be a suitable approach for CT measurement uncertainty assessment. The obtained expanded uncertainties U_95,MPE_ and U_95,VDI_ are comparable (within a 20–25% difference) for a specific workpiece (dog bone). Additionally, for measurands of simplified (dog bone) and 3D complex (dental file) geometries, most of the dimensions present the E_N_ < 1 condition and 2U/T ≤ 0.4 ratio. The application of the 3D Canny method, compared to the local thresholding technique, has proven to provide a more robust and accurate edge definition and, therefore, lower deviations and slightly lower measurement uncertainty due to higher repeatability. The 3D Canny technique allows an improved distinction and determination of the edges, which has been shown to be adequate for the measurement of complex geometries as those presented in this research.

The computation of an exhaustive uncertainty budget in CT metrology is still an issue, and further work on a specific target oriented compensation strategy and uncertainty calculation is needed. In this respect, several assumptions could be considered in order not to overestimate the MPE value of the CT system by analyzing specific common standards (e.g., spheres, gauge blocks, hole plates, etc.), perform measurements on different materials, and perform measurements of inner geometries. Challenges are still present for the complex measurands of the dental file such as the helix angle, and for geometrical characteristics with a critical measurand definition, such as the length of the active cutting edge.

## Figures and Tables

**Table 1:** Uncertainty error contributors included in both approaches: proposed method (MPE) and validation method (VDI/VDE 2630-2.1).

Error Source Description	Proposed Method	Validation Method
Standard uncertainty of calibration	**u_ref_** MPE of the CT estimation	**u_cal_** Task-specific calibration
Standard uncertainty of the measurement procedure (repeatability)	**u_p_**	
Standard uncertainty from the material and manufacturing variations	**u_w_**	
Standard uncertainty associated with the systematic error	**u_b_**	
Uncertainty estimation according to	ISO 14253-2 [20]	VDI/VDE 2630-2.1 [13]

**Table 2:** Dog bone: measurands, description, and nominal and tolerance values.

Measurand	Description	Nominal Value	Tolerance
L	Length	11.80 mm	±0.03 mm
a, c	Length	3.00 mm	±0.03 mm
b	Length	1.50 mm	±0.02 mm
d	Length	1.35 mm	±0.02 mm
A, B, C, D, E, F	Thickness	1.00 mm	±0.02 mm

**Table 3:** Dog bone: micro-CT scanning parameters.

Parameter	Unit	Value
Voltage	kV	80
Current	µA	95
Voxel size	µm	8
Detector matrix		2300 × 3498
Detector pixel size	µm	23.8
Data binning		1 × 1
Frames averaged		7
Exposure time	ms	3000
Magnification		0.3
Increment angle	deg	0.4
No. of views		900

**Table 4:** Dog bone (DB1): Uncertainty contributors and expanded uncertainty (U95, k = 2) according to ISO 14253-2 and MPE estimation, obtained by the CT system with both surface extraction techniques used.

Measurand (DB1)	L Left [µm]		a Left [µm]		b Right [µm]		E Thickness [µm]	
Technique	CT1	CT2	CT1	CT2	CT1	CT2	CT1	CT2
u_ref_	4.39	4.55	3.58	3.77	3.44	3.64	3.39	3.59
u_p_ (u_w1_)	1.72	0.64	1.62	0.51	1.81	0.65	1.79	0.64
u_w2_	0.074	0.074	0.019	0.019	0.010	0.010	0.006	0.006
u_b1_	1.28	1.28	0.32	0.32	0.17	0.17	0.11	0.11
u_b2_	0.92	0.93	0.92	0.93	0.92	0.93	0.92	0.93
**U_95,MPE_**	**9.9**	**9.7**	**8.1**	**7.9**	**8.0**	**7.6**	**7.9**	**7.5**

**Table 5:** Dog bone (DB1): Uncertainty contributors and expanded uncertainty (U95, k = 2) according to VDI/VDE 2630-2.1, obtained by the CT system with both surface extraction techniques used.

Measurand (DB1)	L Left [µm]		a Left [µm]		b Right [µm]		E Thickness [µm]	
Technique	CT1	CT2	CT1	CT2	CT1	CT2	CT1	CT2
u_ref_	2.59	2.59	2.72	2.72	2.53	2.53	2.73	2.73
u_p_ (u_w1_)	1.72	0.64	1.62	0.51	1.81	0.65	1.79	0.64
u_w2_	0.074	0.074	0.019	0.019	0.010	0.010	0.006	0.006
u_b1_	1.28	1.28	0.32	0.32	0.17	0.17	0.11	0.11
u_b2_	0.92	0.93	0.92	0.93	0.92	0.93	0.92	0.93
**U_95,VDI_**	**7.0**	**6.2**	**6.6**	**5.9**	**6.5**	**5.6**	**6.8**	**5.9**

**Table 6:** Dog bone: Percentage of E_N_ < 1 values calculated for all CT measurements, obtained with both calibration procedures (MPE and VDI) and using two surface extraction techniques (CT1, CT2).

Measurand	% of E_N_ < 1 (U_95,MPE,CTi_)		% of E_N_ < 1 (U_95,VDI,CTi_)	
Technique	CT1	CT2	CT1	CT2
L	87.5%	87.5%	75.0%	75.0%
a_left_ and c_right_	12.5%	25.0%	12.5%	0.0%
a_right_ and c_left_	100%	100%	100%	100%
b	75.0%	100%	75.0%	87.5%
d	87.5%	87.5%	75.0%	87.5%
A, B, C, D, E, F	87.5%	95.8%	79.2%	95.8%

**Table 7:** Dental file: measurands, description, and nominal and tolerance values.

Measurand	Description	Nominal Value	Tolerance [40]
La	Length (active cutting part)	16 mm	±0.5 mm
Dn (n = 0, …, 13)	Diameter (variable)	D0 = 0.25 mm to D6	±0.02 mm
		D7 = 0.60 mm to D13	±0.04 mm
Pn (n = 1, …, 11)	Helix pitch (variable)	(*)	(*)
Hn (n = 1, …, 10)	Helix angle (variable)	(*)	(*)

(*) Nominal value and tolerance for helix pitch and helix angle are not specified.

**Table 8:** Dental file: micro-CT scanning parameters.

Parameter	Unit	Value	Parameter	Unit	Value
Voltage	kV	90	Data binning		3 × 3
Current	µA	80	Frames averaged		5
Voxel size	µm	28	Exposure time	ms	3000
Detector matrix		2300 × 3498	Magnification		2.5
Detector pixel size	µm	23.8	Increment angle	deg	0.4
			No. of views		900

**Table 9:** Dental file: Uncertainty contributors and expanded uncertainty (U95, k = 2) according to ISO 14253-2 and MPE obtained by the micro-CT system with both surface extraction techniques used.

Measurand	La [µm]		D11 [µm]		P6 [µm]		H6 (*) [deg]	
**Technique**	CT1	CT2	CT1	CT2	CT1	CT2	CT1	CT2
u_ref_	4.87	4.87	3.37	3.37	3.44	3.44	0.14	0.12
u_p_ (u_w1_)	2.34	0.70	3.15	0.70	7.12	8.05	0.24	0.14
u_w2_	0.012	0.012	0.001	0.001	0.001	0.001	3.4 × 10^−4^	2.8 × 10^−4^
u_b1_	0.22	0.22	0.01	0.01	0.02	0.02	4.4 × 10^−4^	4.0 × 10^−4^
u_b2_	1.65	1.50	1.65	1.50	0.98	0.98	0.040	0.034
**U_95,MPE_**	**11.3**	**10.3**	**9.8**	**7.5**	**16.0**	**17.7**	**0.6**	**0.4**

(*) The helix angle uncertainty was estimated applying the error propagation law (GUM [19]).

**Table 10:** Dental file: Percentage of E_N_ < 1 values calculated for all CT measurements and obtained with the proposed calibration alternative (MPE) using two surface extraction techniques (CT1, CT2).

Measurand	% of E_N_<1 (U_95,MPE,CTi_)	
Technique	CT1	CT2
La	50.0%	25.0%
D0 to D6	71.4%	78.6%
D7 to D13	88.9%	85.2%
Helix pitch	90.0%	95.0%
Helix angle	47.5%	72.5%

**Table 11:** Dental file: Percentage of 2U/T ≤ 0.4 values calculated for all CT measurements and obtained with the proposed calibration alternative (MPE) and using two surface extraction techniques (CT1, CT2), and for the OCMM measurements.

Measurand	2U/T ≤ 0.4 (U_95,MPE,CTi_)		2U/T ≤ 0.4 (U_95,OCMM_)
Technique	CT1	CT2	OCMM
La	100%	100%	100%
D0 to D6	57.1%	71.4%	100%
D7 to D13	85.7%	100%	100%

Helix pitch and helix angle tolerances are not specified.
